# Recombinant nAG (a Salamander-Derived Protein) Decreases the Formation of Hypertrophic Scarring in the Rabbit Ear Model

**DOI:** 10.1155/2014/121098

**Published:** 2014-03-30

**Authors:** Mohammad M. Al-Qattan, Mervat M. Abd-Al Wahed, Khalid Hawary, Ahmed A. Alhumidi, Medhat K. Shier

**Affiliations:** ^1^Department of Surgery, King Saud University, PO Box 18097, Riyadh 11415, Saudi Arabia; ^2^College of Medicine Research Center, King Saud University, PO Box 18097, Riyadh 11415, Saudi Arabia; ^3^Department of Pathology, King Saud University, Riyadh, Saudi Arabia

## Abstract

nAG (newt-Anterrior Gradient) protein is the key mediator of regrowth of amputated limbs in salamanders. In a previous work in our lab, a new *nAG* gene (suitable for humans) was designed and cloned. The cloned vector was transfected into primary human fibroblasts. The expression of *nAG* in human primary fibroblasts was found to suppress collagen expression. The current study shows that local injection of recombinant nAG reduces scar hypertrophy in the rabbit ear model. This is associated with lower scar elevation index (SEI), lower levels of collagen I & III, higher levels of MMP1, and a higher degree of scar maturation in experimental wounds compared to controls.

## 1. Introduction

Hypertrophic scars are pathological scars characterized by elevation, itching, and pain [1]. They are usually seen following delayed wound healing of injured skin in burns, trauma, and surgery [2]. Patients with large areas of hypertrophic scarring have cosmetic, psychological, and functional problems [1].

Although there are several options available for treating hypertrophic scars, there is no single modality that results in normalization of the affected skin. Large areas (such as following burns) are usually treated by a combination of pressure garments and silicone sheets [3]. Small hypertrophic scars are usually treated by repeated intradermal injections of steroids [4]. All treatment modalities of hypertrophic scars are associated with side effects and, hence, the prevention or suppression of the formation of these scars is arising as a new strategy. Several research studies continue to search for agents that “prevent” or “decrease” the formation of hypertrophic scars [5–8].

Regrowth of amputated limbs of salamanders represents a unique form of tissue regeneration. nAG (newt-Anterior Gradient) is the key mediator of this limb regeneration [9]. In previous work we documented the inhibitory effect of* nAG* gene on collagen synthesis. The new* nAG* gene (suitable for humans) was designed, synthesized, and cloned. The cloned vector was then transfected into primary human fibroblasts. The expression of* nAG* in human fibroblasts was found to suppress the expression of collagen with or without transforming growth factor-beta1 (TGF-*β*1) stimulation [10]. The current study investigates the effect of recombinant nAG on decreasing hypertrophic scarring in the rabbit ear model. We show that local injection of recombinant nAG reduces scar hypertrophy in that model and this is associated with reduced collagen I and collagen III deposition as well as a higher degree of scar maturation compared to controls.

## 2. Materials and Methods

### 2.1. Synthesis and Preparation of Human Recombinant nAG Protein

Synthesis and structure of* nAG* gene/protein were described in our previous work [10]. Human recombinant nAG protein was synthesized by Genscript Company (Piscataway, NJ, USA) by using mammalian transient protein expression system (HEK 293-6E cells, mammalian expression vector pTT5). The protein was obtained from culture supernatant, with one step purification by Hi Trap Chelating HP 1 mL Column. The protein purity was 80% and obtained as lyophilized powder. 100 *μ*g of recombinant nAG was dissolved in 53 mL of physiological saline and 53 mg of bovine serum albumin as a stabilizer. The individual doses (to be injected into the experimental model) were stored at −80°C.

### 2.2. The Hypertrophic Scar Model

The hypertrophic scar model using the rabbit ear is a well-established model in the literature and has been verified by several authors [5–8]. The created wound is known to epithelialize in 14 days and an established hypertrophic scar is known to appear at day 28. In studies that aim to show effect of treatment of established hypertrophic scars, the treatment is commenced at day 28. In studies (including our study) that aim to test the decrease of formation of hypertrophic scars (i.e., prevention of scarring), the treatment is commenced at day 14 (i.e., after epithelialization of the wound).

### 2.3. Surgical Procedure

Five white New Zealand rabbits weighing 1.5–2 kg were obtained and single-housed, with food and water provided ad libitum. The animals were anaesthetized with intramuscular ketamine (40 mg/kg) and Xylazine (4 mg/kg). Cleaning of the ears was done with iodine. No local anesthesia was used. One wound was created in each ear (one experimental wound and one control wound). The wound was created down to bare cartilage on the ventral surface of the ear using a 7 mm punch biopsy. A magnifying loupe is used to ensure removal of the epidermis, dermis, and perichondrium in each wound. Haemostasis was obtained by applying pressure with cotton balls.

Wounds were dressed with normal saline-soaked gauze and left to heal spontaneously.

Experimental wounds were injected daily into the wounds (under the new epithelium) with 100 *μ*L solution containing 100 nM of recombinant nAG protein. Control wounds were also injected daily in a similar way with 100 *μ*L of the same solution without nAG. Daily injections started at day 14 and ended at day 27 after wounding in both experimental and control wounds.

### 2.4. Assessment

Assessment was done in experimental and control wounds at day 28 after wounding. Three parameters were assessed: scar elevation index, scar maturation (thickness and alignment of collagen fibers) by histology, and quantitative determination of mRNA of collagen I, collagen III, and MMP-1 (Matrix Metalloproteinase-1).

#### 2.4.1. Scar Elevation Index (SEI)

SEI was used for morphometric analysis as previously described [11]. The maximum protuberant part of the scar was measured by using calipers and the SEI was calculated as the ratio of total wound area tissue height to the area of normal tissue below the scar. A SEI of 1 indicates no formation of hypertrophic scarring, whereas an index >1 indicates hypertrophic scar formation.

#### 2.4.2. Histology

Each scar was bisected at the maximum hypertrophy. One half of each scar was used for histology and the other half for RNA extraction. For histological examination, the specimen was fixed in 4% paraformaldehyde, embedded in paraffin, and sectioned. Sections were stained with Masson's trichrome stain. Scar maturation was assessed by examining collagen fiber thickness and alignment.

#### 2.4.3. RNA Extraction and Quantitative Determination of mRNA of Collagen I, Collagen III, and MMP-1 Using Real-Time PCR

The scar tissue was homogenized by using tissue Lysa LT (Qiagen, Dusseldorf, Germany). Total RNA was then extracted from the homogenized tissue by using RNeasy protect mini kit (Qiagen). Reverse transcription was then performed using a rotor-gene multiplex PCR kit (Qiagen). Specific primers and TaqMan probes for rabbit collagen I, collagen II, and MMP-1 genes were designed using the integrated DNA technology software (Table 1). The TaqMan probes had fluorescent reporter dyes (FAM and HEX) attached to the 5′ end and a quencher (BHQ1) at the 3′ end. The house-keeping gene GAPDH was used as a normalizing gene. Thermal cycling conditions were as follows: an initial reverse transcription step for 15 min at 50°C, incubation at 95°C for 5 min to activate hot star DNA polymerase, and then 40 cycles at 95°C for 15 sec, 60°C for 15 sec. Acquiring on green and yellow channels occurred in the annealing/extension step. Relative quantification of collagen I, collagen II, and MMP-1 expression was obtained by using ΔΔ*c*_*t*_ relative quantification method in software of rotor-gene Q5plex (Qiagen). Gene expression of interest was measured in triplicate in three different real-time PCRs.

### 2.5. Statistical Analysis

SPSS version 19 statistical package was used for data analysis. SEI and RNA extraction data were expressed as mean ± SD. Normality and homogeneity of the variables were checked first.

The differences between the values from the experimental versus the control groups were analyzed using the independent sample *t*-test (for SEI) or the nonparametric Mann-Whitney test (for RNA extraction data). *P* < 0.05 indicated a significant difference between the two groups.

## 3. Results

### 3.1. Scar Elevation Index

**Figure 1:**
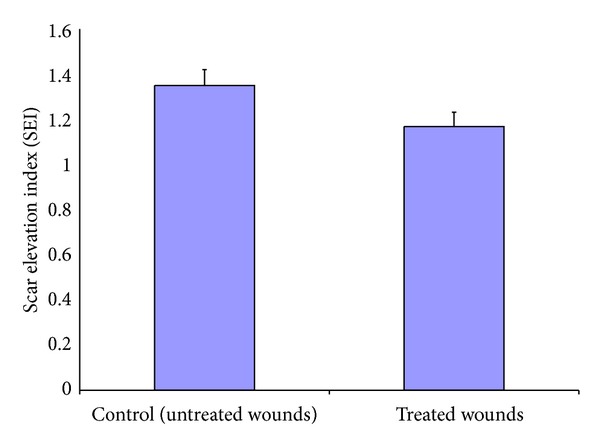
The degree of scar hypertrophy is reflected by SEI. After treatment of wounds with recombinant nAG for 13 days after complete epithelialization, SEI was measured for treated and untreated wounds. There was significant decrease in SEI in treated wounds: 1.168 ± 0.063 compared to the untreated wounds 1.35 ± 0.07 (*P* = 0.003).

At day 28 after wounding, there was significantly more hypertrophic scarring in the control group compared to the experimental group (*P* = 0.003). The mean ± SD scar elevation index of control wounds was 1.35 ± 0.07 compared to 1.17 ± 0.063 in experimental wounds (Figure 1).

### 3.2. Histology

**Figure 2:**
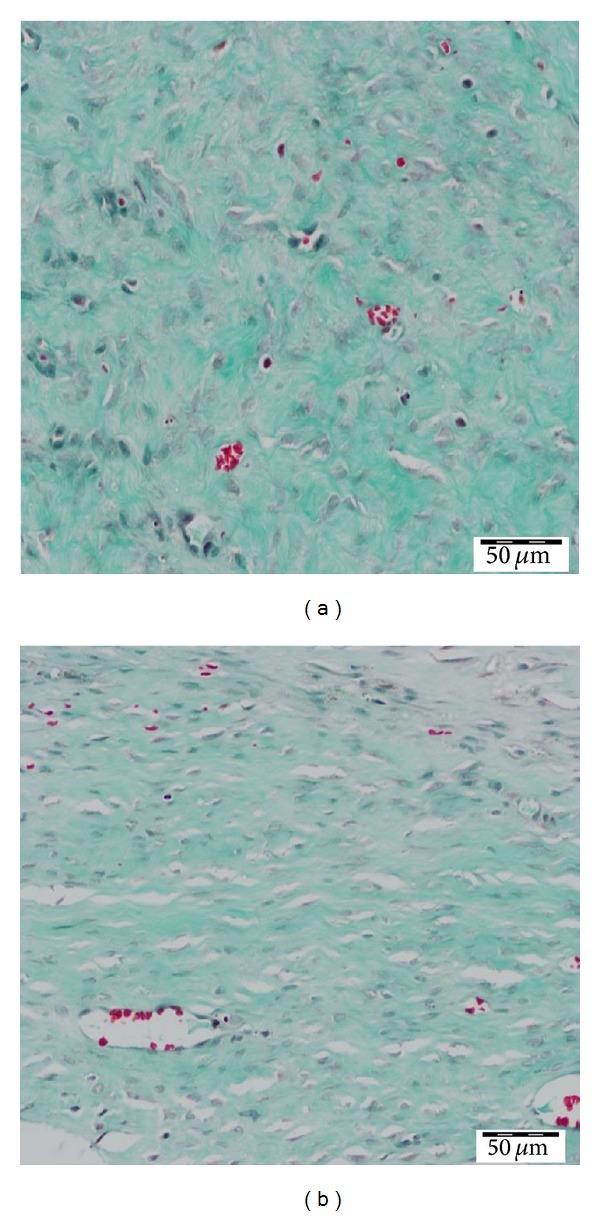
Masson trichrome staining. (a) Collagen fibers in control wounds are thick and disorganized (magnification 40x). (b) Collagen fibers in experimental wounds are thin and organized parallel to each other (magnification 40x).

At day 28 after wounding, there was histological evidence of a higher degree of scar maturation in the experimental wounds (Figure 2). Masson's trichrome staining showed thick, dense, and disorganized collagen fibers in the control wounds (Figure 2(a)), whereas the collagen fibers were thinner and arranged more regularly and parallel in the experimental wounds (Figure 2(b)). The control wounds (Figure 2(a)) also showed more extracellular matrix than the experimental wounds (Figure 2(b)). However, the increased number of small blood vessels was also observed in experimental wounds.

### 3.3. Real-Time PCR Analysis

**Figure 3:**
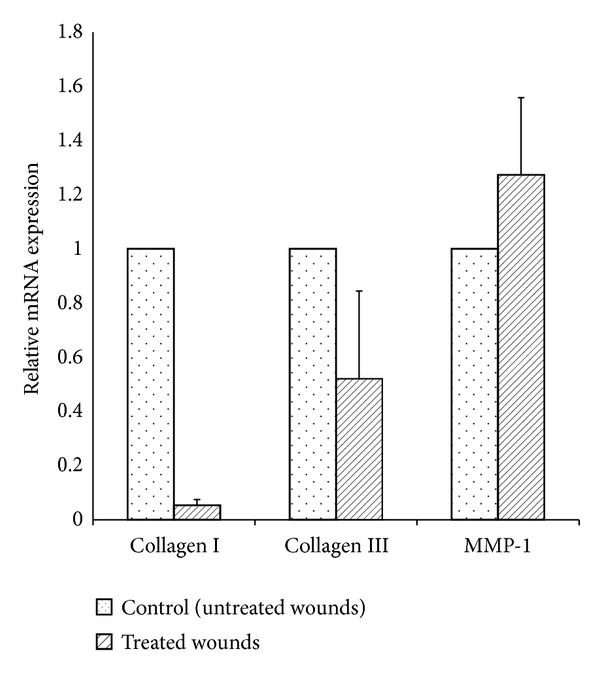
Quantitative real-time PCR measuring relative mRNA expression level of collagen I, collagen III, and MMP-1 in nAG treated wounds and untreated wounds. Total RNA was reverse-transcribed and target genes expression was measured in multiplex, one-step RT-PCR by using TaqMan probes with FAM and HEX reporter dyes and BHQ1 quencher. Relative mRNA expression was related to the reference gene, GAPDH. The relative expression of collagen I was decreased by 95% (*P* < 0.05), collagen III expression was decreased by 48% (*P* < 0.05), and MMP-1 expression was increased by 27% (*P* > 0.05) in nAG treated wounds compared to untreated wounds.

Quantitative determination of mRNA of collagen I, collagen III, and MMP-1 of experimental and control wounds was done at day 28 after wounding. Compared to controls, experimental wounds showed decreased collagen I by 95%, decreased collagen III by 48%, and increased MMPI by 27% (Figure 3). Using the Mann-Whitney test, the decrease of collagen I and collagen III in experimental wounds was statistically significant (*P* < 0.05). However, the increase in MMP1 in experimental wounds compared to control wounds did not reach statistical significance (*P* > 0.05).

## 4. Discussion

Our study introduces a new agent (the recombinant nAG protein) that may be considered to be used for suppression of formation of hypertrophic scarring. The recombinant nAG is a novel protein and, hence, there are no similar studies in the literature. Therefore, we are unable to compare our results to others. Compared with most previous agents described previously in the literature [5–8], the nAG seems to be an attractive agent since it has multiple effects such as suppression of collagen synthesis, enhancement of collagen degradation, and acceleration of wound maturation.

In our previous work, using transfected human fibroblasts, nAG suppressed collagen III more than collagen I [10]. In the rabbit, collagen suppression was more pronounced for collagen I (95% decrease) than for collagen III (48% decrease). This is interesting because it correlates with the well-known differences between the collagen content of human versus rabbit skin [8]. In normal human skin, the content of collagen I is higher than that of collagen III and the opposite is true for normal rabbit skin [8]. Wound maturation is delayed in hypertrophic scarring. Our model demonstrated histological evidence of a higher degree of maturation in experimental wounds because collagen fibers were less dense and more organized in experimental compared to control wounds (Figure 2). The presence of increased number of small vessels in experimental wounds may indicate that nAG-induced inhibition of extensive collagen disposition may be associated with increased neovascularization. Hence, further studies are required to also investigate scarring after cessation of nAG treatment. Furthermore, the systemic effect of nAG was not assessed in the current study and this will be required before investigating the effectiveness of nAG in preventing hypertrophic scars in humans.

Another interesting finding in our study is regarding the results of the MMP1 levels. MMP1 is the main enzyme that degrades collagen I and collagen III in scars [12]. In our study, by using the nAG, the MMP1 levels were higher in the experimental group than in the control group, although the difference did not reach statistical significance. In other studies, such as the one using topical oleanolic acid to suppress hypertrophic scars in the rabbit ear model, the MMP1 levels were higher in the control group than in the experimental group [8]. Although this is difficult to explain, it may be due to the variable effects of the agent used on MMP1 and the biofeedback regulating mechanisms in wounds in vivo since a higher production of MMP1 is induced in wounds with higher collagen content [8].

## 5. Conclusion

Recombinant nAG decreases the formation of hypertrophic scarring in the rabbit ear model. This is associated with lower SEI, lower levels of collagen I and collagen III, higher levels of MMP1, and a higher degree of scar maturation in experimental wounds compared to controls.

## Figures and Tables

**Table 1:** Primers and probes used for real-time PCR.

Gene	Gene accession number	Primer and probe sequence	Amplicon size (bp)
Rab. COL1 F.P.	NM_001195668 XM_002713800	CCCAACCAAGGATGCACTAT	126
Rab. COL1 Probe		5′-FAM, TCTCTACTGGCGAAACCTGCATCC, BHQ1-3′	
Rab. COL1 R.P.		CTTGGCCTTGGAGCTCTTATAC	

Rab. COL3 F.P.	S83371.1	CATTGGCCCTGTTTGCTTT	110
Rab. COL3 Probe		5′-FAM, AAACCAACCTCTTCCTGAAGCCC, BHQ1-3′	
Rab. COL3 R.P.		CACTTGTACTGGTTGACAAGATTAG	

Rab. MMP1 F.P.	NM_001171139	GCAGAATGAGCTACCAGGATAC	89
Rab. MMP1 Probe		5′-FAM, AGGACATTCACAGCTCCTTTGGCT, BHQ1-3′	
Rab. MMP1 R.P.		CAGAAACAGCAGCGTCAATATG	

Rab. GAPDH F.P.	NM_001082253.1	ATGGCCTCCAAGGAGTAAGA	149
Rab. GAPDH Probe		5′-HEX, CAGTCCCACCACGCTGAGAATCTG, BHQ1-3′	
Rab. GAPDH R.P.		CTGGGATGGAAACTGTGAAGAG	
